# Khat chewing and health related quality of life: cross-sectional study in Jazan region, Kingdom of Saudi Arabia

**DOI:** 10.1186/1477-7525-12-44

**Published:** 2014-04-04

**Authors:** Kamaludin Ahmed Sheikh, Maged El-setouhy, Umar Yagoub, Rashad Alsanosy, Zafar Ahmed

**Affiliations:** 1 Medical Research Center, Jazan University, Jazan, Kingdom of Saudi Arabia; 2 Substance Abuse Research Center, Jazan University, Jazan, Kingdom of Saudi Arabia; 3 Family and Community Medicine, Medical Services Department, Ministry of Defense, Riyadh, Kingdom of Saudi Arabia; 4 University Kebangsaan Malaysia (Nations University of Malaysia), Kuala Lumpur, Malaysia; 5 Department of Community, Environmental and Occupational Medicine, Faculty of Medicine, Ain Shams University, Cairo, Egypt

**Keywords:** Khat chewing, Health related quality of life, FS-36 questionnaire

## Abstract

**Background:**

The chewing of Khat leaves, a natural psychoactive substance is widely chewed in countries of East Africa and the southern Arabian Peninsula, and is reported to be associated with a range of unfavorable health outcomes including khat dependence. The impact of Khat chewing on Health Related Quality of Life is yet to be explored. Aims: to measure and compare the quality of life of the khat chewers and non-khat chewers using a short form health survey (SF36), and to assess factors associated with Khat chewing using SF36 in a sample of adult population in Jazan region, Kingdom of Saudi Arabia.

**Methods:**

A total of 630 participants from two independent male populations of khat chewers and non-khat chewers were recruited into a cross-sectional survey study. A self administrative survey based on the SF-36 questionnaire was used to collect data on measures of health-related quality of life (HRQoL). Socioeconomic data of the respondents were also collected for detailed analysis. Data analysis include: descriptive statistics, reliability tests (Cronbach’s alpha and intraclass correlation coefficient), and bivariate analysis (Chi square and Mann–Whitney U-test) to compare HRQoL of Khat chewers and non-Khat chewers.

**Results:**

The odds of being a khat chewer were higher in respondents with a lower socioeconomic status. The SF-36 scores were significantly lower in all domains for respondents with khat chewing, indicating that non-khat chewers had higher health perceptions compared with those chewing khat. The overall mean score of HRQoL for non-khat chewers was 92.7% (SD 5.53) compared with 63.5% (SD 21.73) for the khat chewing group. The study had shown good internal consistency and reproducibility across the eight subscales of SF-36 questionnaire (α 0.74-0.95). The Mann–Whitney U-test showed a significant difference between khat chewers and non-khat chewers (P < 0.001).

**Conclusions:**

This study measured and compared the quality of life of khat chewers and non-khat chewers using a generic health survey (SF36). The study had shown that khat chewing is associated with lower quality of life (HRQoL) and lower socioeconomic status. However in future a more refined SF36 developed especially for Khat chewers can provide more useful information.

## Background

Khat (Catha edulis Forsk) is a natural psychoactive substance, which has been chewed (usually chewed) for many years in Ethiopia, East Africa, and the southern Arabian Peninsula [1-3]. With the recent globalization, khat chewing has spread with African and Arabian immigrants to various Asian [4] and European [5-9] countries, and to Australia [10-12], as well as to the United States [13]. The users of khat in these new countries are predominantly immigrants from the khat chewing countries, but khat chewing is slowly spreading to the native population of the host countries [9]. The main active (addictive) substances, cathinone and cathine (Schedule I and IV drugs, respectively), are contained in khat leaves, and are considered prohibited substances as per the schedule list of the United Nation’s International Convention on Psychotropic Substances [14-16]. The stimulant effect of khat is more potent (because of cathinone) when the leaves are still fresh, and 48 h after being harvested, the stimulant elements in khat convert into a less active potent (cathine), which also has an amphetamine-like action [3,17-19]. In short, the current literature suggests that the stimulant and health effects of khat are related to cathinone in khat, though other components of khat are still under research.

Khat is a mild psycho-stimulant that increases alertness, enhances mood, and reduces the need to sleep [7,11,20]. Khat is chewed for its euphoric and stimulating effects [11,21], but it has many negative effects on different body systems. Khat chewing had been associated with gastrointestinal problems, such as mouth ulcers, inflammation of the esophagus and stomach, gum disease, jaw problems, and constipation [22]. Khat chewing is also associated with hypertension [22-25], coronary vasoconstriction, and myocardial infarction [24,26-28]. Moreover, khat chewing is associated with increased susceptibility for stroke and early death [23]. Khat stimulates locomotor and stereotypic behavioral activity, and can induce seizures [29]. Khat chewing can impair driving ability, and therefore increases road traffic accident rates [30]. Despite the apparent adverse effect of khat chewing on the khat chewer’s health, no studies have evaluated the quality of life among khat chewers. This study attempts to fill that gap.

Khat chewing is highly prevalent in Yemen (82% among men and 43% among women) [31]. Jazan province of Saudi Arabia lies at the far southwestern corner of the country, adjacent to Yemen. This could be the main reason why khat chewing is widely prevalent in Jazan province as well. Khat chewing has been a major public health problem in the Jazan province in the Kingdom of Saudi Arabia (KSA) for a long time. In Jazan province, the prevalence of khat chewing is 49% among the general male population but it can increase to 62% in rural areas of Jazan [32-37]. Khat chewing is prohibited in Saudi Arabia and within the Jazan region, but its chewing is still increasing at an alarming rate, especially among the younger population in high schools and institutions of higher learning [35]. The prevalence of khat chewing among male high school students and university students in Jazan province is estimated at 21% and 38%, respectively [34]. To manage this problem, the Saudi Government has been trying to control the expansion of chewing, cultivation, and trade of khat. In this regard, the Saudi Government has enacted many policies and rules on the prohibition of khat chewing. However, even these strict government policies have failed to eradicate the chewing of khat in Jazan province [33].

To assess health-related quality of life (HRQoL), we used the short form 36 (SF-36) questionnaires, which is a standardized generic instrument, commonly used to measure health status [36]. The main objectives of this study were: 1) To measure and compare the quality of life of khat chewers and non-khat chewers using a short form health survey (SF36), and 2) To assess and explore factors associated with Khat chewing using SF36 in a sample of adult population in Jazan region, Kingdom of Saudi Arabia.

## Methods

### Study design, sampling, and sample size

This was a cross-sectional survey study of male khat chewers and non-khat chewers who attended primary healthcare centers in Jazan province in the Kingdom of Saudi Arabia. The inclusion criteria for the participants of the study were male ≥18 years old attending selected primary healthcare centers located in the Jazan region, no disabilities that would prevent understanding the questionnaire, no consumption of substances other than khat and tobacco, and no associated co-morbidities and history of chronic diseases. Because of cultural sensitivities and the domination of men in khat chewing, we excluded the female population from the study sample.

A total of 630 participants from two independent populations of khat and non-khat chewers were selected to participate in this study from March 2 to July 31, 2012. The questionnaires were designed to be self-administered, but in some cases a trained research assistant read the questionnaire to illiterate subjects and recorded their responses. The sample required to compare the two population means of khat chewers and non-khat chewers was 608 (304 subjects in each group) using the following formula [37,38]:

$$n=\frac{2*{\left(z_{1}{-}_{\alpha /2}+{z_{1-}}_{\beta }\right)}^{2}}{d^{2}}$$

where *α* = 0.05 and *β* = 0.20. The values of *z*_1 - *a*/2_ and *z*_1 - *β*_ are 1.96 and 0.84, respectively, 2(*z*_1 - *a*/2_ + *z*_1 - *β*_)^2^ = 15.68, and  *d*^2^ = *δ*/*σ* (δ is the difference between the two population means and σ is the standard deviation of the measurements; both δ = 5 and σ = 22 were found from a pilot test). Therefore, the sample size required in each group was calculated to be 304 patients and the two samples scenario required a total of 608 subjects. We allowed 5% for incomplete data or missing values in the data, and the required sample size was increased to be 630 participants. The sampling was done at in two stages. In the first stage a random sample of four out of 13 primary healthcare centers were selected. In the second stage, within each selected medical center, we invited all patients who visited the clinic during the research duration to participate in our study, and collected the information required until we obtained the sample size required. A sample of 630 (94%) who completed the questionnaires were used for the analysis. 40 (6%) patients refused to participate (27 [4%]) in the study or provided incomplete data and (13 [2%]) were excluded.

### Ethical approval

This study was reviewed and accepted by the ethics committee at Jazan University. All of the participants of the study signed a written informed consent and agreed willingly and voluntarily to participate in the study.

### Study instrument

The SF-36 is currently the most commonly used health status measure worldwide [38]. This instrument is a standardized generic instrument developed by the RAND Corporation (USA). The SF-36 provides a simple descriptive summary and a single index value for health status [39]. The SF-36 consists of 36 questions divided into eight domains: physical functioning (PF), bodily pain (BP), social functioning (SF), general health (GH), role-physical (RP), role-emotional (RE), vitality (VT), and mental health (MH). The item scores in each of the factors can be coded, summed, and transformed into a scale ranging from 0 (the worst imaginable health state) to 100 (the best imaginable health state). This instrument is easy to administer, not time consuming, available in Arabic translation, and valid (Cronbach’s alpha value was 0.65) [39,40].

**Figure 1:**
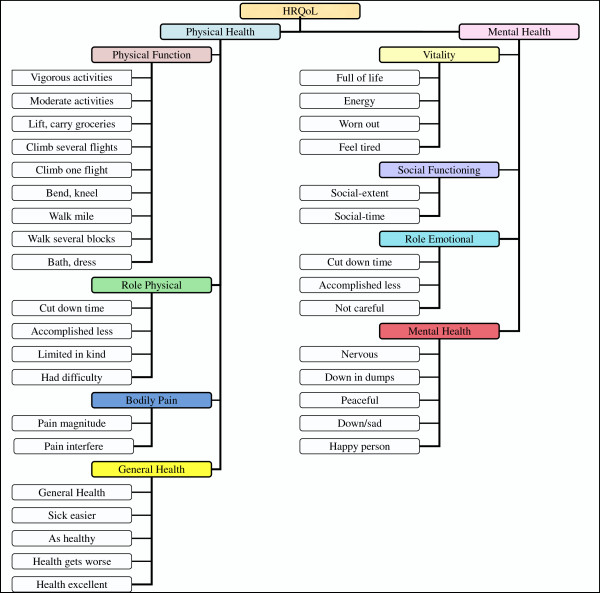
SF-36 conceptual framework.

The SF-36 questionnaire was used to measure the HRQoL of khat chewers in the Jazan region. Even though the SF-36 questionnaire is a self-administered instrument, six research assistants were recruited and trained by the research team to help patients having difficulty understanding the questions. These trained research assistants were available during the research period at every clinic to assist the study participants in case the participants have any problem in understanding the context of the questions. These research assistants are suppose to provide guidance only and do not influence the participants opinion in any way. Figure 1 shows the conceptual framework used with the SF-36 instrument in this study. The eight components of the SF-36 score were classified into two main groups: physical health and mental health questions. The physical health section includes questions on PF, RP, BP, and GH, whereas the mental health section questions are on MH, RE, SF, and VT.

### Statistical analysis

Descriptive statistics for each dimension reported for the SF-36 questionnaire included the mean, standard deviation, coefficient of variation, skewness, kurtosis, and range for the scales. The demographic variables of the respondents were also reported with the age of the respondents in both groups categorized into four groups: 18–24 years, 25–34 years, 35–44 years, and ≥ 45 years. The odds ratio test was used to identify the association between the demographic characteristics of the respondents and their status of khat chewing. Mann–Whitney (MW) and Equality of mean tests with 95% CI were used to compare the differences in HRQoL between khat chewers and non-khat chewers. MW test is a non-parametric test used when the dependent variable cannot be assumed to be normally distributed. The Kolmogorov-Smirnov test was used to evaluate the normality of the study data collected using the SF-36 instrument. The kurtosis and skewness statistics of the two samples indicated non-normally distributed data. The coefficient of variance of the two groups reflected that the data had an acceptable degree of variance and homogeneity [41,42]. Cronbach’s alpha and the intraclass correlation coefficient (ICC) were used to evaluate the reliability and consistency of the SF-36 instrument. The ICC approach used was two-way mixed effect model with consistency type. A reliability of 0.6 is considered as acceptably reliable, while 0.8 or higher is considered a good reliability; and for the ICC a value greater than 0.3 is acceptable. Finally, a pilot test of 60 and 56 respondents on each of the two independent groups of khat chewers and non-khat chewers was performed, respectively, to calculate the δ (difference between the two population means) and σ (standard deviation of the measurements). All statistical analyses were performed using SPSS 16.0. A statistical value of P < 0.05 was taken as significant.

## Results

### Characteristics of the sample

The SF-36 questionnaire was distributed to 670 participants from two independent populations of khat and non-khat chewers. Finally, a sample of 630 (94%) who completed the questionnaires were used for the analysis. The participants completed the questionnaire in 10–30 min, with 80% completing the questionnaire in 15 min or less. A total of 40 (6%) patients either refused to participate in the study or provided incomplete data (27 [4%] and 13 [2%] patients, respectively).

The descriptive statistics for the SF-36 data collected for both groups are given in Table 1. The Kolmogorov-Smirnov test for normality provided evidence that the distributions of the two populations of khat chewers and non-khat chewers were significantly different from each other (P < 0.001); indicating non-normally distributed data. Although the normality assumption is violated, we still interpret the mean values and a coefficient of variation of the data, reporting the mean for interpretation purposes is required, even when the distribution is not normal. In the sample of non-khat chewers, the average values for the eight components of the SF-36 were between 88.71 (GH) and 96.47 (SF), whereas for the sample of khat chewers, the average values of the eight components of the SF-36 were between 52.83 and 78.60. Non-khat chewers had higher mean scores for all the eight subscales of the SF-36 compared with khat chewers (Table 1). The overall mean score of HRQoL for the non-khat chewer group was higher (p < 0.001) than that in the khat chewer group (92.7% vs 63.5%).

**Table 1:** Characteristics of the sample and features of score distributions for the SF-36 scale

**Description**	**PF**	**RP**	**BP**	**GH**	**MH**	**RE**	**SF**	**VT**
**No. of items**	**10**	**4**	**2**	**5**	**5**	**3**	**2**	**4**
K-S test	4.582^a^	4.143^a^	3.386^a^	4.263^a^	6.972^a^	6.534^a^	6.972^a^	8.885^a^
Mean								
Non-Khat	89.05	90.32	92.30	88.71	94.41	95.77	96.47	94.21
Khat cons	72.70	66.03	78.60	71.49	56.94	53.44	56.03	52.83
SD								
Non-Khat	15.64	18.45	12.93	12.94	14.34	12.33	9.11	13.53
Khat cons	30.96	37.34	27.33	24.97	35.30	43.61	39.51	31.62
CV								
Non-Khat	0.18	0.21	0.14	0.15	0.15	0.15	0.10	0.14
Khat cons	0.40	0.57	0.35	0.35	0.62	0.82	0.71	0.60
Skewness								
Non-Khat	-2.092	-2.661	-2.393	-1.056	-2.705	-4.206	-3.324	-2.372
Khat cons	-0.654	-0.634	-1.569	-0.739	0.083	-0.180	-0.209	-0.081
Kurtosis								
Non-Khat	5.449	8.832	7.003	0.515	6.359	20.002	11.330	4.834
Khat cons	-1.034	-1.099	1.624	-0.687	-1.539	-1.708	-1.473	-1.479
Range	0 – 100	0 – 100	0 – 100	0 – 100	0 – 100	0 – 100	0 – 100	0 – 95

*Abbreviations*: *PF* Physical Functioning, *RP* Role-Physical, *BP* Bodily Pain, *GH* General Health, *MH* Mental Health, *RE* Role-Emotional, *SF* Social Functioning, *VT* Vitality, *CV* Coefficient of Variance, *K-S* Kolmogorov-Smirnov test, *a.* Asymp. Sig. (two-tailed) - Kolmogorov-Smirnov Z.

### Demographic variables in relation to khat chewing status

Cross-tabulation analysis was performed to determine the association of some selected demographic variables versus the status of khat chewing. Four demographic factors were selected for this analysis; marital status, employment status, age classified into groups, and education. Table 2 shows the cross-tabulation results of socio-demographic variables and the status of khat chewers and non-khat chewers. Among the respondents from the khat chewer group, 148 (39.2%) khat chewers were married, whereas 167 (66.5%) khat chewers were not married. In contrast, among the respondents from the non-khat chewer group, 230 (60.8%) non-khat chewers were married, whereas 85 (33.7%) non-khat chewers were not married. This result showed that the odds of being a non-khat chewer being married were 67.2% higher than the reference group of unmarried.

**Table 2:** Comparisons of socio-demographic characteristics and khat chewing status

	**Non-khat Chewers (%)**	**Khat Chewers (%)**	**Total (%)**	**OR (95% CI)**
Marital status				
Not-Married	85 (33.7)	167 (66.5)	252 (40.0)	Reference
Married	230 (60.8)	148 (39.2)	378 (60.0)	0.328 (0.235, 0.457)
Total	315 (50.0)	315 (50.0)	630 (100)	
Employment				
Self-employed	94 (80.3)	23 (19.7)	117 (18.6)	Reference
Government employed	84 (72.4)	32 (27.6)	116 (18.4)	1.557 (0.845, 2.869)
Private employed	62 (63.9)	35 (36.1)	97 (15.4)	2.307 (1.246, 4.272)
Unemployed	33 (37.9)	54 (62.1)	87 (13.8)	6.688 (3.566, 12.542)
Student	42 (19.7)	171 (80.3)	213 (33.8)	16.640 (9.436, 29.34)
Total	315 (50.0)	315 (50.0)	630 (100)	
Age (years)				
18 – 24	63 (34.8)	118 (65.2)	181 (28.7)	Reference
25 – 34	49 (32.0)	104 (68.0)	153 (24.3)	1.133 (0.717, 1.790)
35 – 44	101 (62.3)	61 (37.7)	162 (25.7)	0.322 (0.207, 0.501)
≥ 45	102 (76.1)	32 (23.9)	134 (21.3)	0.167 (0.101, 0.276)
Total	315 (50.0)	315 (50.0)	630 (100)	
Education				
No formal schooling	18 (21.7)	65 (78.3)	83 (13.2)	Reference
Primary school	17 (20.2)	67 (79.8)	84 (13.3)	1.091 (0.518, 2.300)
Secondary school	73 (41.5)	103 (58.5)	176 (27.9)	0.391 (0.214, 0.713)
Diploma	116 (65.2)	62 (34.8)	178 (28.3)	0.148 (0.081, 0.271)
Degree	91 (83.5)	18 (16.5)	109 (17.3)	0.055 (0.026, 0.113)
Total	315 (50.0)	315 (50.0)	630 (100)	

With regard to employment status, the majority of self-employed, government-employed, and privately employed respondents were reported to be non-khat chewers, while the majority of unemployed and students were reported to be khat chewers. The odds of being a khat chewer among government employees was 1.56 (95% confidence interval [CI]: 0.845, 2.869) times higher than the reference group of the self-employed respondents, but this was not significant. The odds of being a khat chewer among the unemployed and student participants was 6.68 (95% CI: 3.566, 12.542) times and 16.640 (95% CI: 9.436, 29.34) times higher, respectively, than the reference group of self-employed.

Older respondents were more likely to be non-khat chewers than younger respondents. The probability of khat chewing among the age group of 25–34 years was identical to that of the reference age group (odds ratio: 1.13; (95% CI: 0.717, 1.790). The older age groups of 35–44 years and ≥ 45 years had a lower probability of being a khat chewer compared with the reference age group, 0.322 (95% CI: 0.207, 0.501) and 0.167 (95% CI: 0.101, 0.276), respectively. Finally, the respondents with an education level of secondary school or higher were less likely to be khat chewers than respondents with a lower education level. The odds of being a khat chewer/non-khat chewer among respondents with a primary education were not different from the respondents with no formal schooling (odds ratio: 1.091; 95% CI: 0.518, 2.300).

### Comparison of mean SF-36 scores between khat and non-khat chewers

The mean differences of the physical health perceptions were between 13.71 for bodily pain (BP) and 24.29 role-physical (RP); whereas, the mean differences of the mental health perceptions were between 37.47 Mental Health (MH) and 42.33 Role Emotional (RE). On average, the health gap (mean difference) between khat and non-khat users for mental health perceptions was larger than the health gap for physical perceptions. This indicates that khat chewers complain more about mental and social problems rather than physical problems (Table 3). The Mann–Whitney U-test showed that non-khat chewers had significantly higher mean scores of HRQoL across all of the domains of the SF-36 than khat chewers (P < 0.001).

**Table 3:** Mean comparison of HRQoL scores of Khat chewers vs non-Khat chewers

		**Mean**	**Mean differ.**	**95% CI of the Difference**		**Mann–Whitney Test**
**Description of SF-36 domains**						
				**Lower**	**Upper**	
Physical Functioning (10 items)						
Khat Chewer		72.70	16.35	12.512	20.187	38435^b^
Non-Khat Chewer		89.05				
Role Physical Limitation (4 items)						
Khat Chewer		66.03	24.29	19.668	28.904	32044^b^
Non-Khat Chewer		90.32				
Bodily Pain (2 items)						
Khat Chewer		78.60	13.71	10.370	17.042	34232^b^
Non-Khat Chewer		92.30				
General Health (5 items)						
Khat Chewer		71.49	17.22	14.103	20.341	28899^b^
Non-Khat Chewer		88.71				
Mental Health (5 items)						
Khat Chewer		56.94	37.47	33.269	41.677	21010^b^
Non-Khat Chewer		94.41				
Role Emotional (3 items)						
Khat Chewer		53.44	42.33	37.237	47.419	22240^b^
Non-Khat Chewer		95.77				
Social Functioning (2 items)						
Khat Chewer		56.03	40.44	35.934	44.939	20876^b^
Non-Khat Chewer		96.47				
Vitality (4 items)						
Khat Chewer		52.83	41.38	37.601	45.161	9822.0^b^
Non-Khat Chewer		94.21				

*Abbreviations*: *PF* Physical functioning, *RP* Role-physical, *BP* Bodily pain, *GH* General health, *MH* Mental health, *RE* Role emotional, *SF* Social functioning, *VT* Vitality, *CV* Coefficient of variance, *b* Asymp. Sig. (two-tailed) - Mann–Whitney U.

Table 4 shows that all of the eight subscales of the SF-36 instrument demonstrated high internal consistency, as measured by Cronbach’s alpha, which approximately exceeded 0.7 (range, 0.73–0.95). The table also shows that the ICC ranged from 0.312 to 0.769, which is acceptable and indicates consistency of the data [41]. Generally, respondents from the sample of khat chewers had higher ICC values and Cronbach’s alpha than respondents who were non-khat chewers.

**Table 4:** Internal consistency of the SF-36 for khat and non-khat chewer patients

**Dimensions**	**# Items**	**Khat Chewers**		**Non-khat Chewers**	
		**Cronbach’s alpha**	**Intra-class correlation**	**Cronbach’s alpha**	**Intra-class correlation**
Physical function (PF)	10	0.950	0.654	0.867	0.395
Role physical (RP)	4	0.782	0.473	0.727	0.400
Body pain (BP)	2	0.774	0.632	0.735	0.581
General health (GH)	5	0.738	0.361	0.694	0.312
Mental health (MH)	5	0.911	0.672	0.737	0.360
Role-emotional (RE)	3	0.871	0.693	0.895	0.739
Social function (SF)	2	0.852	0.742	0.719	0.561
Vitality (VT)	4	0.930	0.769	0.746	0.423

## Discussion

HRQoL is a widely used instrument for assessing the physical and psychosocial impact of diseases and conditions, and this measure has led to a better understanding of populations’ health and conditions. The SF-36 instrument is one of the most widely used instruments to measure the HRQoL in medical research. Our results showed that the SF-36 was relatively quick and easy to use for the assessment of quality of life of khat chewers. All of the participants managed to complete the SF-36 instrument in a timely manner. The primary finding of this study was that the quality of life score of khat chewers was significantly lower than that of non-khat chewers. In addition, we also found that the physical health perception scores of khat chewers were much higher than the mental health perception scores. This finding indicates that physical harm from khat is less than societal and mental harm, which entails huge disability and discomfort [33,43]. There are several possibilities that can explain the differences between groups, e.g. 1) people with lower QoL are more likely to become khat chewers (this is a variant of the popular self-medication hypothesis, e.g. Khantzian, 1997 [44], or of the functional use hypothesis, e.g. Boy, Marsden & Strang 2001 [45]), 2) an independent third variable (e.g. burden or socioeconomic status) explains why people have lower QoL and higher khat use or 3) Some chemical ingredients in khat might have caused chewers to report lower levels of quality of life.

We found that khat chewers were characterized by low socioeconomic conditions; similar to other types of substance abuse, but this was not the case in non-khat chewers (Table 2). The variables of education, age, employment status, and marital status were negatively associated with khat chewing. This suggested that the odds of being khat chewers were minimal among older respondents, and those with a good employment status and higher education level. However, respondents less than 35 years, who were unmarried, had inactive employment and a lower education level, had higher odds of being khat chewers. More than 70% of khat chewers were younger than 35 years, and the unemployment rate was 62% among khat chewers. This is in accordance with the opinions reported by other studies, which have reported that excessive khat use is associated with reduced productivity and risk of unemployment because khat reduces motivation of work and increases the occurrences of work absenteeism [8,46,47].

Moreover, khat chewing was reported as a main source of family problem that reduces the quality of life of the spouses; this could be explained by the following reasons: First, khat is usually chewed in group sessions and takes long hours (6 hours per session) of chewing with friends and colleagues. This habit routinely causes the chewer to neglect their families and consequently prompts conflict with the spouse [48]. Second, money spent on khat purchases can also cause conflict between spouses. Third, sleeping problems that are often associated with khat chewing severely affects working hours and consequently reduces family income. Finally, the emotional instability, mood swings and the bad temper associated with khat chewing can cause the chewer to be violent and aggressive towards the spouse [48].

Socioeconomic variables play a large role in shaping the characteristics of individuals. People with a higher socioeconomic status are less likely to be involved in regular use of substances and drugs compared with people with lower socioeconomic status [49-51]. Some studies have reported that migrant khat chewers in Europe and North America are culturally isolated and are in miserable socioeconomic situations [52,53]. In the general literature, socioeconomic status is often characterized as a root cause of health inequalities and health risk factors; primary prevention activities, such as increasing law enforcement efforts and community-based interventions focusing on social networking and improving the socioeconomic conditions, may help change khat chewing behavior or prevent potential khat users from indulging in khat chewing [54]. Law enforcement interventions are among the most frequent policies to fight with substance abuse habits; and in our opinion this intervention can at least stop the spread of khat chewing from lower socio-economic class to the higher socio-economic class.

Previous studies have reported that substance users (i.e., alcohol and opiates) have lower HRQoL than the general population [55-60]. The Mann–Whitney test indicated that the mean rank scores of the two groups were significantly different from each other. The HRQoL scores of khat chewers in this study were relatively low (indicating a worse perception of HRQoL) compared with the non-khat chewers, especially for VT, RE, and SF. The difference in mean between the two groups gets smaller in the dimensions of BP, PF, and GH [60]. This indicates that societal and mental health burden due to khat chewing is comparatively higher than the physical harm due to khat chewing; this can be explained by that khat is sometimes associated with mental health problems, such as depression, paranoia, hallucination, manic behavior, hyperactivity, and some other mental disorders [61-66].

The relatively close scores of physical health of khat and non-khat chewers showed less impairment of khat chewing in the physical and functioning dimensions compared with emotional and social function. These findings are consistent with results of other similar studies on alcoholics [56,67]. Finally, the Cronbach alpha coefficients indicated good internal consistency, with values generally higher or equal to 0.70, and these results were similar to those described in other studies [55,57], but were lower than those recommended in the literature (i.e., between 0.85 and 0.95) [42,67]. These findings indicated that the SF-36 instrument was a good tool for the purposes of this study.

### Limitations and future research implications

Similar to other self-reported questionnaires, this study was subject to recall bias of patients and selection bias of medical centers. Additionally, instead of using a generic instrument (SF-36 questionnaire) it may be more appropriate to use a specifically designed instrument for the effects of khat. The sample representativeness is questionable; meaning any inferences from this study population to the whole population was not appropriate. The HRQOL was not verified in this study with medical records, which could have added value to results. Tobacco smoking was not investigated; this could have confounded the results. The social desirability in this study may have been eliminated through using the self-administered questionnaire though we do not know the number of participants who self-administered questionnaires and those who provided data through face to face interviews. The implication of this study for future research with respect to validation of these study findings and answering relevant questions that include the health economic impacts (cost analysis) of khat chewing is appropriate to be reported within the discussion. Future research is to consider data from female khat chewers as well.

Measuring the quality of life is a broad topic and it is impossible to form a definite conclusion as to why non-khat chewers had higher rates of physical and mental health compared with their khat chewing counterparts. Some chemical ingredients in khat might have caused chewers to report lower levels of quality of life. Another possibility is that users with a low quality of life use khat more frequently. These possibilities need to be explored further, and could be a focal point for future studies.

## Conclusions

This study assessed the perceptions of quality of life of khat chewers and compared them with non-khat chewers according to their educational level, age, marital status, and employment status, using the SF-36 survey tool. Evaluation of quality of life might be useful for providing more information about health disparities among society, and will draw attention to the need for allocating more resources to vulnerable populations. Our study shows that non-khat chewers have higher health perceptions and a higher socioeconomic status compared with khat chewers. These findings indicate that khat is associated with lower quality of life (HRQoL) and lower socioeconomic status. Therefore, intervention measures are indicated to manage this problem. Primary prevention activities, such as increasing law enforcement efforts and community-based interventions focusing on social networking and improving the socioeconomic conditions, may help change khat chewing behavior or prevent potential khat users from indulging in khat chewing.

Finally, this study identified several research gaps, which are of interest for future research. It will also extend the scope for the researcher to understand such type of social chronic problem practically. In this way, the study will become instrumental to comprehend khat users the burden of khat chewing on their health and livelihood development. One key recommendation for future researchers might be to further study on causal mechanisms exist behind the association of low QoL and higher khat use. The relationship between HRQoL and treatment outcomes in khat addicts should also be investigated. In summary, the study findings indicate the need for health promotion among khat users, as suggested by the lower socio-economic status and lower HRQoL status of the khat chewers. All our study findings are consistent with the results of earlier studies on substance abuse and addiction. However in future a more refined SF36 developed especially for Khat chewers can provide more useful information.

## Competing interests

The authors have no financial competing interests to declare.

## Authors’ contributions

KA is the principal investigator of the project, who designed the research protocol, interpreted the results, supervised the study, and wrote the manuscript. ME conducted data collection, and participated in writing the manuscript and manuscript revision. UY performed data entry and statistical analysis, and wrote the manuscript. RE performed data management and critically edited the manuscript. ZA assisted in statistical analysis, interpretation, and manuscript revision. This final manuscript was read and approved by all authors.
